# Targeting the Microenvironment in Esophageal Cancer

**DOI:** 10.3389/fcell.2021.684966

**Published:** 2021-08-26

**Authors:** Lei Wang, Huiqiong Han, Zehua Wang, Litong Shi, Mei Yang, Yanru Qin

**Affiliations:** ^1^Department of Oncology, The First Affiliated Hospital of Zhengzhou University, Zhengzhou, China; ^2^State Key Laboratory of Esophageal Cancer Prevention and Treatment, Zhengzhou University, Zhengzhou, China

**Keywords:** esophageal cancer, tumor microenvironment, vascular endothelial growth factor, PD-1/PD-L1, cancer associated fibroblasts

## Abstract

Esophageal cancer (EC) is the eighth most common type of cancer and the sixth leading cause of cancer-related deaths worldwide. At present, the clinical treatment for EC is based mainly on radical surgery, chemotherapy, and radiotherapy. However, due to the limited efficacy of conventional treatments and the serious adverse reactions, the outcome is still unsatisfactory (the 5-year survival rate for patients is less than 25%). Thus, it is extremely important and urgent to identify new therapeutic targets. The concept of tumor microenvironment (TME) has attracted increased attention since it was proposed. Recent studies have shown that TME is an important therapeutic target for EC. Microenvironment-targeting therapies such as immunotherapy and antiangiogenic therapy have played an indispensable role in prolonging survival and improving the prognosis of patients with EC. In addition, many new drugs and therapies that have been developed to target microenvironment may become treatment options in the future. We summarize the microenvironment of EC and the latest advances in microenvironment-targeting therapies in this review.

## Introduction

Esophageal cancer (EC) is one of the most common malignancies and is a major global health challenge. In 2018, new cases of EC accounted for 3.2% of the total cancer cases and EC-related deaths accounted for 5.3% of the total cancer deaths (Bray et al., 2018; Wang L. et al., 2021). EC consists of two main pathologies—esophageal squamous cell carcinoma (ESCC) and esophageal adenocarcinoma (EAC). ESCC is the most common, mainly in East Asia and Africa, while EAC is mainly prevalent in North America and Europe (Arnold et al., 2015; Torre et al., 2015). Geographical distribution of EC is associated with diet and genetics (Smyth et al., 2017). More than half of patients with EC are often at an advanced stage when first diagnosed, and extensive metastasis makes radical surgery, which is currently the only cure for EC, impossible (Ohashi et al., 2015). At present, the treatment for advanced EC include chemotherapy, radiotherapy, and a few targeted drugs. Although great strides have been made in diagnosis and treatment, the 5-year survival rate for patients with advanced EC is still very poor; no more than 25% (Enzinger and Mayer, 2003). The high mortality rate in EC patients indicates that better treatment methods and targets are needed.

The “seed and soil” hypothesis proposed by Paget (1989) is the prototype for the tumor microenvironment (TME). Subsequent complementary studies have found that TME is composed of a variety of different cells and proteins, including immune cells, extracellular matrix (ECM), and tumor blood vessels (Polyak et al., 2009). In addition, TME has several important characteristics, including hypoxia, acidosis, chronic inflammation, and immunosuppression, which are associated with tumor proliferation, migration, apoptosis, immune evasion, and angiogenesis (Sung and Chung, 2002; Whiteside, 2008). Moreover, TME is not invariant, but involves constant remodeling of cells and their secretions to make them more suitable for tumor survival (Han et al., 2020). This is partly responsible for the development of resistance to conventional treatments (Luan et al., 2021). All these indicate that the “soil” of EC is important for tumor survival and may be an avenue for overcoming neoplastic disease.

Cells in the microenvironment have better gene stability, suggesting that TME-targeted treatments may have better effects and lower chances of drug resistance. To date, the most popular therapies targeting TME in EC include antiangiogenic therapy (anti-VEGF) and immunotherapy (PD-1/PD-L1 inhibitors) (Kojima et al., 2020; Yang Y. M. et al., 2020; Zhang B. et al., 2020). Both monotherapy and combination therapies further improved treatment efficacy and prolonged survival in patients with EC. In addition, anti-inflammatory reoxygenation combined with radiotherapy or photodynamic therapy, tumor vaccine, blocking microenvironment signal transduction, and other new therapies prevent occurrence and improve the prognosis of EC (Huang and Fu, 2019; Liu J. et al., 2020; Yamamoto and Kato, 2020; Yang Y. M. et al., 2020). Moreover, many cells and factors in the microenvironment can be used as important indicators to judge the prognosis of EC (Lin et al., 2016; Han et al., 2020). This review summarizes the EC microenvironment and related targeted therapies.

## Suppressing Inflammation Prevents Esophageal Cancer

The relationship between inflammation and cancer has been a key focus of research, and long-term inflammatory stimulation is an important inducer of EC (Coussens and Werb, 2002). The EC microenvironment is filled with a variety of pro-inflammatory cytokines and inflammatory substances, all of which are closely associated with tumor occurrence, proliferation, and migration (Bhat et al., 2021). Systematic activation of inflammatory pathway signals promotes the progression of EC. Nuclear factor-kappa B (NF-κB) consists of a family of structurally related transcription factors (Zhang et al., 2019), and its elevated expression is considered a marker of inflammation-induced tumorigenesis (Karin et al., 2002; Izzo et al., 2006). In addition to the NF-κB signaling pathway, interleukin-6 (IL-6)/STAT3 signaling pathway was also found to be upregulated in EC (Wang et al., 2004; Groblewska et al., 2012a). IL-6 is a cytokine that signals by binding to gp130 *via* its receptor, IL-6Rα, to trigger downstream pathways and activate important molecules such as Ras-MAPK, SHP2, PI3K, STAT1, and STAT3. Activation of these pathways gives tumor cells the ability to survive in a highly toxic inflammatory environment and inhibits the effects of immunotherapy (Karin et al., 2002; Hodge et al., 2005).

There are several differences in the inflammatory microenvironment of ESCC and EAC. ESCC is the most common pathological type of EC in East Asia (Smyth et al., 2017), and several well-known carcinogenic factors, such as alcohol and smoking (Enzinger and Mayer, 2003; Rustgi and El-Serag, 2014), cause chronic irritation and subsequent inflammation of the esophageal epithelium through direct toxic effects and reactive oxygen species (ROS) production (Radojicic et al., 2012; Kubo et al., 2014). Epidemiological studies of high-risk populations in China have found that frequent consumption of superheated foods also increases the incidence of ESCC, which is thought to damage the esophageal epithelium and lead to increased inflammation (Shen et al., 2020). Thus, there is little doubt that chronic inflammation is a risk factor for ESCC. Barrett’s esophagus is a precancerous lesion of EAC in which chronic gastroesophageal reflux (GERD) causes esophageal epithelial cells to be replaced by goblet cells (Dvorak et al., 2007). Gastric acid reflux directly damages the esophagus and promotes ROS production. Direct injury can trigger sonic hedgehog (SHH) signaling between the damaged epithelium and adjacent stroma, leading to intestinal metaplasia (Wang et al., 2010). Infiltrating inflammatory cells also produce high quantities of ROS to promote epithelial cell transformation and the production of ROS directly leads to DNA damage, causing tumor-initiation mutations (Poulsen et al., 1998; Farhadi et al., 2002). Epidemiological studies have linked obesity to EC (Kamat et al., 2009; Rustgi and El-Serag, 2014). Obesity is in fact a systemic inflammation and a metabolic disorder, which is thought to play an important role in the origin of malignant diseases (Bianchini et al., 2002). There are several mechanisms that can explain the association between obesity and EC, including increased incidence of GERD, increased secretion of proinflammatory adipocytokines in the serum, causing insulin and insulin-like growth factor secretion disorder, and leptin (Eusebi et al., 2012; Greer et al., 2012; Mokrowiecka et al., 2012). In addition to obesity, microbes are also important factors. The imbalance in the oral and intestinal flora can lead to inflammation and gastroesophageal reflux. Based on analysis of high-risk populations, this imbalance is mainly manifested as a decrease in gram-positive bacteria and an increase in gram-negative bacteria (Yang L. et al., 2012; Walker and Talley, 2014).

Normal cells are more likely to mutate in an environment filled with inflammatory cells and cytokines, leading to the development of tumors (Coussens and Werb, 2002). Therefore, anti-inflammatory therapy is a very effective preventive measure. Primary prevention of EC involves improving lifestyle, that is, keeping away from the risk factors for inflammation, including avoiding smoking, consuming moderate quantities of alcohol, and maintaining a healthy weight. For patients with esophagitis or Barrett’s esophagus, secondary prevention includes medication with proton pump inhibitors (PPIs) and prokinetics (e.g., Domperidone and Itopride). Anti-reflux surgery is also a form of primary prophylaxis. Some meta-analyses and cohort studies have shown that patients with Barrett’s esophagus who were treated with PPIs had a lower incidence of dysplasia and EAC compared with those patients who were not treated with PPIs (Nguyen et al., 2009; Kastelein et al., 2013). Several drugs have also been shown to inhibit the production of inflammatory factors, thereby inhibiting inflammation. Curcumin, which is found in the household spice turmeric, can inhibit acid-induced IL-6 and IL-8 production by inhibiting activation of the MAPK and PKC signaling pathways, as well as NF-κB (Rafiee et al., 2009). This drug is expected to treat esophagitis caused by GERD. In addition to prevention, inhibition of inflammation can increase the sensitivity of radiation and chemotherapy *in vivo* and *in vitro* and forms one approach for comprehensively treating EC (Li et al., 2018; Liao et al., 2020).

## Anti-Angiogenesis Is a Classic Microenvironment-Targeting Therapy

Angiogenesis plays an essential role in the development of most solid tumors, including EC, by delivering oxygen and nutrients to the tumor. Tumor angiogenesis is regulated by a variety of angiogenic factors such as vascular endothelial growth factor (VEGF), hepatocyte growth factor (HGF), transforming growth factor-beta (TGF-β), and hypoxia-inducible factor-1 (HIF-1) (Ladeira et al., 2018). Hypoxia, acidosis, and nutritional deficiency can all upregulate the expression of VEGF and promote angiogenesis. Distant metastasis through blood vessels is an additional pathway for tumor progression. As early as Folkman (1971) speculated that blocking tumor blood vessels could inhibit tumor growth. Anti-angiogenic therapies, particularly VEGF inhibitors, have gradually improved following years of research and have played an important role in clinical treatment.

**FIGURE 1:**
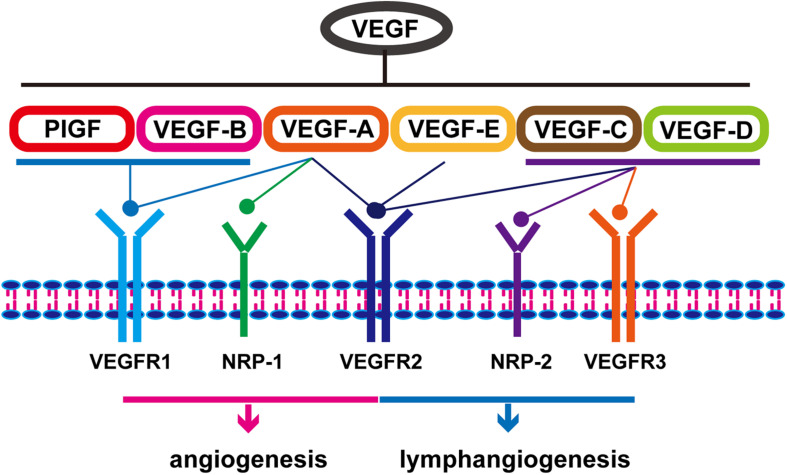
Classification and function of vascular endothelial growth factors.

The key mediator of angiogenesis is VEGF, including VEGF-A/B/C/D/E and placental growth factor (PIGF) (Roskoski, 2007). As shown in Figure 1, activation of VEGF/VEGFR and VEGF/NRP pathways not only promote the proliferation of vascular endothelial cells and accelerate angiogenesis but also play an important role in promoting lymphangiogenesis (Ding et al., 2006). VEGFR is also expressed in tumor cells. The binding of VEGF to VEGFR triggers multiple downstream signaling pathways, such as ERK1/2 and PI3K/Akt, to promote cell proliferation (Olsson et al., 2006; Chrzanowska-Wodnicka et al., 2008). Therefore, the VEGF/VEGFR signaling pathway is an effective target for the treatment of EC. A variety of VEGF/VEGFR inhibitors have been developed, including anlotinib, apatinib, sorafenib, sunitinib, ramucirumab, and bevacizumab (Table 1). Of these, anlotinib, apatinib, sorafenib, and ramucirumab have been shown to have clinical benefits in patients with EC during clinical trials (Wilke et al., 2014; Xu et al., 2014; Janjigian et al., 2015; Moehler et al., 2016; Cunningham et al., 2017; Liu G. et al., 2020; Yang Y. M. et al., 2020; Huang et al., 2021). The positive effect of Endostar combined with radiotherapy and chemotherapy in the treatment of ESCC has been reported and similar clinical trials are ongoing (Xu et al., 2014). Anlotinib and apatinib are included in the Chinese Society of Clinical Oncology (CSCO)-EC guidelines as important treatments for EC. Ramucirumab is also included in the National Comprehensive Cancer Network (NCCN) guidelines for the treatment of gastroesophageal junction (GEJ) adenocarcinoma (Bang et al., 2020). In addition to monotherapy, combination with chemotherapy, immunotherapy, or radiotherapy can result in a better curative effect. Researchers successfully treated a patient with advanced ESCC using apatinib in combination with the PD-1 inhibitor camrelizumab (Yan et al., 2020). Li et al. (2019) showed that the combined use of apatinib and docetaxel significantly prolonged patients’ survival and had controllable side effects. Currently, additional studies are exploring the use of combinations of anti-angiogenesis therapy and traditional therapies such as radiotherapy and chemotherapy. On the other hand, tumor blood vessels are structurally and functionally abnormal. This abnormality makes effective drug delivery become difficult and creates an abnormal microenvironment (e.g., hypoxia) that reduces the effectiveness of radiotherapy and chemotherapy. Researchers have found that using anti-angiogenic drugs can induce normalization of blood vessels, then making patients more sensitive to chemotherapy (Batchelor et al., 2007).

**TABLE 1:** Vascular endothelial growth factor inhibitors applied to esophageal cancer.

Molecule	NCT	Target	Strategy	Type	Result	References
Bevacizumab	NCT00450203	VEGF-A	Combination	EAC	Failure	Cunningham et al., 2017
Ramucirumab	NCT01170663	VEGFR2	Combination	GEJ	Success	Wilke et al., 2014
	NCT01246960	VEGFR2	Combination	EAC/GEJ	Failure	Yoon et al., 2016
	NCT02314117	VEGFR2	Combination	GEJ	Failure	Fuchs et al., 2019
Sunitinib	NCT00702884	VEGFRs	Monotherapy	GEJ	Failure	Wu et al., 2015
	NCT00730353	VEGFRs	Combination	EC/GEJ	Failure	Schmitt et al., 2012
Sorafenib	NCT00917462	VEGFRs	Monotherapy	GEJ	Success	Janjigian et al., 2015
	NCT00253370	VEGFRs	Combination	GEJ	Success	Sun et al., 2010
Apatinib	NCT03274011	VEGFR2	Monotherapy	ESCC	Ongoing	U.S. National Library of Medicine, 2021a
	NCT03603756	VEGFR2	Combination	ESCC	Success	Zhang B. et al., 2020
	NCT02942329	VEGFR2	Combination	GEJ	Success	Xu et al., 2019
Anlotinib	NCT02649361	VEGFRs	Monotherapy	ESCC	Success	Huang et al., 2021
Endostar	NCT03797625	VEGFs	Combination	ESCC	Ongoing	U.S. National Library of Medicine, 2021b

However, anti-angiogenesis therapy has some limitations. In addition to several manageable side effects such as hypertension, renal dysfunction, thrombosis, and wound-healing complications, anti-angiogenic drugs are suspected of affecting the spread of other chemotherapeutic drugs *in vivo* (Ye, 2016). Using positron emission tomography (PET), Van der Veldt and others observed that anti-angiogenic drugs could inhibit the delivery of cytotoxic drugs to the tumor site (Van der Veldt et al., 2012). This is not consistent with our previous theory that anti-angiogenic therapy induces structural and functional changes in tumor blood vessels that make them more similar to normal blood vessels, leading to increased blood flow and easier access of cytotoxic drugs into tumors (Batchelor et al., 2007). Zhao et al. (2019) found that small doses of apatinib could regulate TME, alleviate hypoxia, and increase the number of T cells at tumor sites, then enhance the efficacy of PD-1/PD-L1 inhibitors. Excessive doses do not have this effect. This theory has not yet been tested in EC, but it shows that adjusting the order and dosage of medication during treatment is necessary to obtain better efficacy. Current research suggests that anti-angiogenesis therapy combined with other treatments can achieve better therapeutic effects; thus, actively developing new angiogenesis inhibitors and exploring additional drug combination regimens is still the main focus of research efforts.

## Maturation of Immunotherapy

**FIGURE 2:**
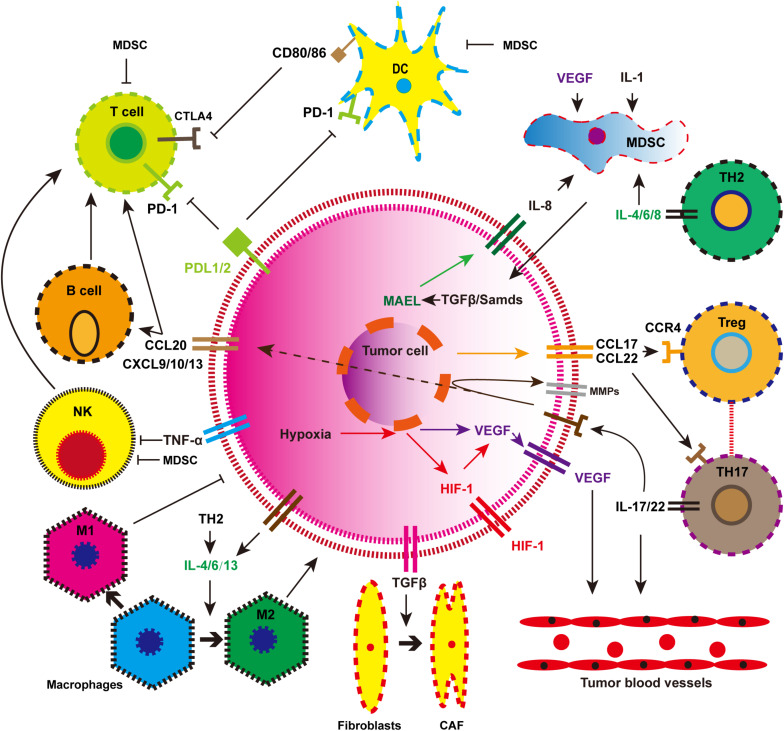
The immune landscape of esophageal cancer. MDSC, myeloid-derived suppressor cell; DC, mature dendritic cell; NK, natural killer cell; Treg, regulatory T cell; M1, tumor suppressor macrophages; M2, tumor-promoting macrophages; CAF, cancer-associated fibroblasts.

Immunotherapy may be the most significant breakthrough in the history of tumor treatment. In-depth study of the immune microenvironment of EC and accurate intervention has become a consensus among most people. The components of the immune microenvironment of EC are complex and diverse. As shown in Figure 2, tumor cells can inhibit the anti-tumor immune response by recruiting a variety of immune cell populations or expressing inhibitory molecular factors (Lin et al., 2016). Smart tumor cells disguise themselves and secrete a variety of cytokines to escape attack by T cells. Immunotherapy suppresses the expression of related pathways or provides immune system-specific tumor antigens that restore the immune system function and eliminates tumor cells. Mainstream immunotherapies include inhibition of immune checkpoints (PD-1/PD-L1), tumor vaccination, and adoptive T-cell therapy. These are described in detail below.

### Immune Checkpoint Blockade

Programmed death-1 (PD-1) is an immune checkpoint for T cells that can deactivate their immune function. Two ligands, PD-L1 and PD-L2, bind to PD-1 receptors, induce PD-1 signal and associated T-cell depletion, and reversibly inhibit T-cell activation and proliferation (Zou et al., 2016). Activation of the PD-1/PD-L1 signaling pathway can inhibit the function of CTL, while inhibition of this signaling pathway can restore T lymphocyte function and enhance the immune response (Chen and Mellman, 2017). Based on this principle, PD-1/PD-L1 blockers were developed for the treatment of tumors and have shown promise in the treatment of multiple malignancies (Topalian et al., 2012).

Programmed death-1 inhibitors such as pembrolizumab and camrelizumab have recently been approved for the treatment of EC following extensive clinical trials. KEYNOTE-181 is a global multicenter, randomized, controlled, open, phase III clinical trial including 628 patients with advanced or metastatic EC. Final experimental results showed that, compared with chemotherapy, pembrolizumab prolonged overall survival when used as a second-line therapy for advanced EC in patients with PD-L1 ≥ 10, with fewer treatment-related adverse events being reported (Kojima et al., 2020). In the latest KEYNOTE-590 study, pembrolizumab combined with cisplatin-fluoropyrimidine chemotherapy as first-line therapy significantly improved overall survival in patients with EC compared with placebo (Smyth et al., 2021). Another PD-1 inhibitor invented in China, camrelizumab, was approved for the treatment of advanced ESCC in 2019 (Zhang B. et al., 2020). In addition to the two PD-1 inhibitors mentioned above, there are multiple PD-1/PD-L1 inhibitors undergoing experimental verification, which will provide more options for immunotherapy in EC patients (Yamamoto and Kato, 2020). Compared with traditional chemotherapy, PD-1/PD-L1 inhibitors have fewer side effects and are more effective.

In clinical practice, most patients do not get better survival outcomes following the administration of PD-1 inhibitors. Therefore, predictive biomarkers are needed to determine whether patients are more likely to respond to PD-1/PD-L1 inhibitors. In KEYNOTE-181, researchers found that, compared with chemotherapy, pembrolizumab significantly increased overall survival in PD-L1-positive patients. This may indicate that PD-L1 expression is a direct biological predictor. Disappointingly, PD-L1 status was not associated with objective response rates (ORR) in Chinese ESCC patients (Huang et al., 2018). Thus, we cannot predict therapeutic effect based only on the expression of PD-L1 in patients. Mismatch repair (MMR) defect, tumor mutation load (TML), and microsatellite instability (MSI) have been identified as predictive biomarkers in non-small cell lung cancer, but their role in EC needs to be validated (Yang H. et al., 2020). Li et al. (2021) recently found that upregulation of Laminin γ2 (Ln-γ2) resulted in worse anti-PD-1 treatment outcome, which could be an effective biological predictor in the future. CAF-derived TGF-β1 signaling leads to T-cell exclusion by increasing the expression of Ln-γ2 in ESCC cells, thereby constructing a protective barrier to the tumor, preventing immune cells from penetrating into tumor parenchyma, and weakening the response to anti-PD-1 therapy. In addition, analysis of 260 patients with ESCC showed that Ln-γ2 is also an independent prognostic predictor. Furthermore, biometric analysis of several serological indicators and a variety of genes including HSPA6, CACYBP, DKK1, EGF, FGF19, GAST, OSM, and ANGPTL3 has also been implicated in having predictive significance (Guo et al., 2020). In fact, it is not very accurate to use a single indicator to predict the efficacy of immunotherapy. Lee and Ruppin found that comprehensive analysis of CD8 T-cell abundance, TML, and PD-1 gene expression can give a more accurate prognosis (Lee and Ruppin, 2019). Whether the comprehensive use of the above indicators can accurately screen out immunotherapy-sensitive populations requires further exploration.

The expression of cytotoxic T-lymphocyte-associated antigen 4 (CTLA4) reduces T-cell activity by inhibiting T-cell receptor (TCR) signaling. A number of studies have shown that overexpression of CTLA4 can block the T-cell cycle, thereby reducing the body’s specific immune function and leading to immune evasion of cancer cells (Krummel and Allison, 1995). Interestingly, CTLA4 is not only expressed by tumor infiltrating immune cells (TIICs) in EC, but is also expressed on cancer cells, which is an important part of tumor cell immune escape (Huang and Fu, 2019). Several studies have proven that CTLA4-targeted therapy can produce good survival benefits and fewer side effects. Currently available drugs for CTLA4 include ipilimumab and tremlimumab, of which tremlimumab has been proven to have a therapeutic effect on EC in clinical trials (Ralph et al., 2010). PD-1 and CTLA4 have different mechanisms for reducing T-cell activation and the combined use of these two immune checkpoint inhibitors may yield better results. This synergistic effect (ipilimumab-nivolumab combination) has been demonstrated in melanoma; it is unproven but promising in EC (Weber et al., 2015).

### Cancer Vaccines

Tumor-testicular antigens (TTA) are the most well-studied tumor-associated antigens (TAA) that are highly expressed in EC, including New York esophageal squamous cell carcinoma 1 (NY-ESO-1), melanoma-associated antigen-A (MAGE-A), TTK protein kinase (TTK), and Cancer-testis antigen 2 (CTAG2; also known as LAGE1) (Huang and Fu, 2019). Cancer vaccines induce an immune response through these specific antigens, stimulating CTLs to recognize and attack tumor cells. Several peptide vaccines are already in clinical trials (Kantoff et al., 2010; Kageyama et al., 2013). Cancer vaccines containing a combination of multiple peptides derived from TTK, lymphocyte antigen 6 complex locus K (LY6K), and insulin-like growth factor-II mRNA binding protein 3 (IMP3) were tested in phase II clinical trials for treatment of advanced EC (Kono et al., 2012). Results demonstrated that vaccine-induced immune responses in patients with advanced ESCC are associated with better outcomes, suggesting that tumor vaccine therapy using multiple epitope peptides as monotherapy may provide clinical benefits for EC patients. Another vaccine is DC vaccine pulsed with peptides. The powerful antigen-presenting function of DC cells enables the body to produce a stronger immune response which kills the tumor. Sadanaga et al. generated autologous DCs *ex vivo* and pulsed them with MAGE-3 peptide (Sadanaga et al., 2001). This was the first report of DC vaccination of EC patients with MAGE-3 peptide. No toxicity was observed *in vivo*, and tumor regression was induced by an immune response to MAGE-3 peptide. At present, tumor vaccines are not formally used in clinical practice, but their strong and specific anti-tumor function requires further study.

### Chimeric Antigen Receptor T-Cell Therapy

Chimeric antigen receptor-T cell therapy refers to the modification of T cells into chimeric antigen receptor (CAR) T cells through genetic engineering to specifically recognize and attack tumor cells. CAR-T cell therapy is more commonly used in hematologic tumors such as leukemia and lymphoma. In recent years, CAR-T cells have been explored as a therapy against solid tumors, including EC (Kiesgen et al., 2018). Studies have shown that ephrin type A receptor 2 (EphA2) and human epidermal growth factor receptor 2 (HER2) are highly expressed in ESCC which are common targets for CAR-T cell therapies (Shi et al., 2018; Yu et al., 2020). Both CAR-T cell therapies have been demonstrated to effectively identify, bind, and destroy ESCC cell lines and release high levels of pro-inflammatory cytokines (Shi et al., 2018; Yu et al., 2020). Kagoya et al. (2018) recently designed a new generation of CAR-T cells with enhanced specificity, persistence, and anti-tumor ability by modifying the previous domain. Based on the CAR-T cell design described above, Zhang H. et al. (2020) designed enhanced MUC1-CAR-T cells for eliminating EC, which were shown to have significant antitumor activity. This enhanced MUC1-CAR-T cells have a longer survival time in mice, which means that they can have sustained anti-tumor ability. The enhanced CAR-T cells seem to be able to overcome the limitations of traditional CAR-T cells. The application of CAR-T cells in solid tumors still has certain limitations, including in the selection of solid tumor-specific antigens and the delivery of CAR-T cells (Akhoundi et al., 2021). Therefore, additional breakthroughs are needed in these areas.

### Oncolytic Viruses

Recently, Challenor and Tucker (2021) reported the case of one patient with Hodgkin’s lymphoma whose tumor disappeared after being infected with SARS-CoV-2. They hypothesized that the SARS-CoV-2 triggered a tumor immune response that allowed T cells to attack cancer cells. This is a special case, but it suggests that viral therapy may be effective. Oncolytic viruses are potential antitumor agents with unique therapeutic mechanisms, including the ability to directly lyse tumors and induce antitumor immunity. Since the first oncolytic virus (*Talimogene laherparepvec*) was approved for the treatment of melanoma, its use has been broadened, including in multiple experiments on EC (Andtbacka et al., 2015). Ma et al. (2012) confirmed that trichostatin enhanced the antitumor activity of oncolytic adenovirus H101 by activating the MAPK/ERK pathway. Another study used radiotherapy in combination with OBP-301, an attenuate type 5 adenovirus with oncolytic potential that contains the human telomerase reverse transcriptase promoter, to regulate viral replication, which is important for the treatment of EC (Kuroda et al., 2010).

## Extracellular Matrix and Signal Transduction Accelerate Tumor Progression

The ECM is an important component of the TME, a network of proteins and glycosaminoglycan (Aguado et al., 2016; Hoshiba and Tanaka, 2016). ECM continues to be remodeled to adapt to the survival and progression of tumors. Easily occurring metastasis is one of the characteristics of malignant tumors and one of the reasons why tumors cannot be cured. Many studies have shown that the dynamic changes in ECM promote tumor metastasis.

### Stromal Components

The most abundant component in ECM is type I collagen, which is secreted by tumor-associated fibroblasts (TAFs) or cancer-associated fibroblasts (CAFs), fills the gaps between cancer cells, and enhances the stiffness of the tumor (Kai et al., 2019). Dense ECM can inhibit the diffusion, penetration, and transportation of therapeutic drugs; thus, ECM becomes an obstacle to drug delivery (Cun et al., 2015; Jena et al., 2016). Another very important molecule is glycoproteins, which are involved in cell-to-cell adhesion and which can be altered to facilitate migration of cancer cells (Palumbo et al., 2020). For example, deletion of E-cadherin, which is responsible for cell-cell adhesion and communication, has been shown to be associated with increased aggressiveness of tumor cells (Canel et al., 2013). Integrins are a family of transmembrane glycoprotein adhesion receptors that regulate extracellular matrix and cellular adhesion. Kwon et al. (2013) inhibited the proliferation and invasion of EC cells by knocking out integrin alpha 6 (ITGA6) *in vitro*, proving that ITGA6 could be a new therapeutic target. So, it is generally believed that the improvement in tumor stiffness and structure is directly associated with tumor invasion.

Remodeling of ECM is dependent on matrix degradable proteolytic enzymes, which mainly include matrix metalloproteinases (MMPs), plasminogen activators, atypical proteinases (e.g., intracellular cathepsin), and glycolytic enzymes (heparinase and hyaluronidase) (Piperigkou et al., 2021). MMPs are characterized by multi-domain zinc-dependent endopeptidases, which play an indispensable role in the continuous remodeling of ECM (Lei et al., 2020). Through ECM remodeling, MMPs regulate the proliferation, migration, and angiogenesis of tumor cells. More than 30 types of MMP have been identified, among which MMP-2 and MMP-9 are the most closely related to EC (Groblewska et al., 2012b). Overexpression of MMP-2 and MMP-9 results in poor prognosis in EC patients due to type IV collagen basement membrane rupture and is associated with advanced tumor stage, local invasion, and metastasis (Groblewska et al., 2012b). MMPs are regulated by their endogenous natural inhibitors (TIMPs), but in EC, this regulation mechanism is abnormal. The decrease in TIMPs expression and the increase in MMPs expression indicate poor prognosis in patients with EC (Groblewska et al., 2012b). Researchers are currently trying to synthesize exogenous MMP inhibitors (MMPIs) that inhibit tumor progression. Thanks to progress in drug technology, MMPIs now have higher specificity and lower toxic and side effects, and their related therapeutic effects have been verified in the treatments of periodontal disease, multiple sclerosis, and gastric cancer and may also be a new target for the treatment of EC (Fields, 2019). Another very important enzyme is lysyl oxidase (LOX), which catalyzes the cross-linking of collagen and elastin. Kalikawe et al. (2019) found that silencing LOX could inhibit the proliferation of ESCC cells and reduce their invasion and migration ability. Understanding the mechanism of these enzymes will benefit our clinical treatment.

**FIGURE 3:**
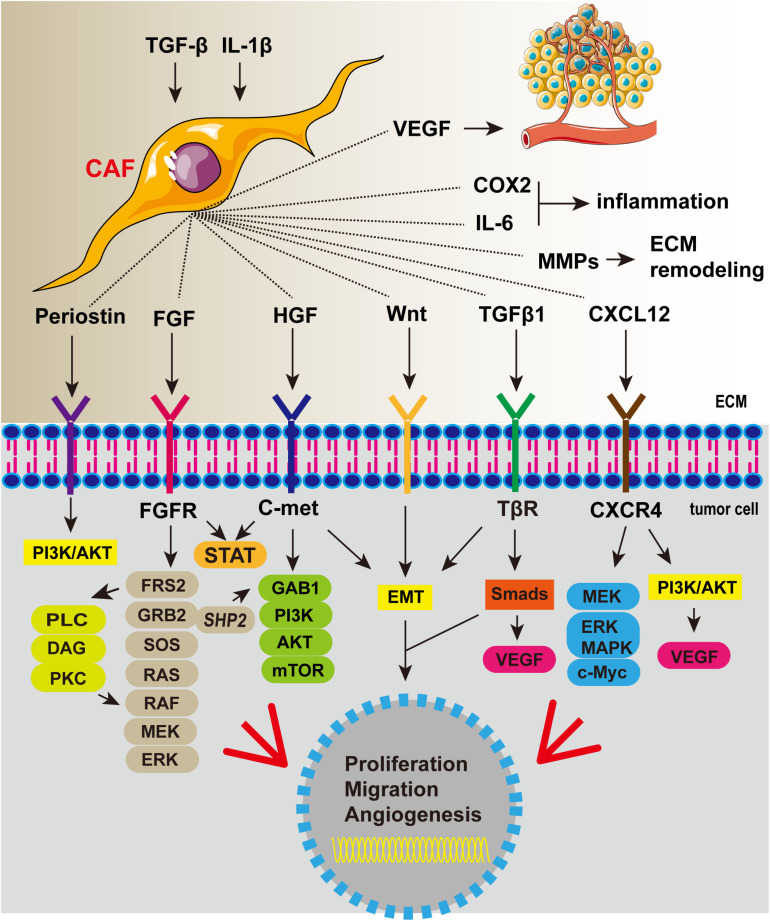
Cancer-associated fibroblasts secrete a variety of cytokines that promote tumor proliferation, invasion, and angiogenesis and aggravate inflammation.

Cancer-associated fibroblast is involved in the development of cancer (Figure 3). In Barrett’s esophagus, reflux of stomach acid can stimulate the production of IL-6 by esophageal fibroblasts and increase inflammation (Rieder et al., 2007). CAFs are activated by cytokines secreted by tumor cells such as TGF-β. More importantly, fibroblast-derived factor can induce esophageal epithelial metaplasia (Eda et al., 2003). CAFs can secrete a variety of important cytokines, including HGF, fibroblast growth factor (FGF), CXCL12, and TGFβ1, which play an important role in promoting the progression of EC. Subsequent studies confirmed that CAFs can also express VEGF, suggesting that it may be involved in EC angiogenesis (Nie et al., 2014). CAFs are also promoters of tumor invasion and metastasis. They produce factors such as Wnt2 that induce epithelial-mesenchymal transformation (EMT) of EC cells and thus increase cell motility (Fu et al., 2011). CAFs are strongly implicated in tumor progression; thus, eliminating CAFs as a way of inhibiting tumor progression may be a good idea. *In vitro* and animal model studies showed that EC cells co-cultured with CAFs were more prone to metastasis and that cell migration reduced after removal of CAFs (Kashima et al., 2019). In recent years, researchers have tried to inhibit tumor progression by eliminating or inhibiting CAFs. For example, near-infrared photoimmunotherapy (NIR-PIT) was proposed by Mitsunaga et al. (2011) as a new cancer treatment method using highly selective monoclonal antibody (mAb)-photosensitizer conjugate (APC). CAF elimination using CAF-targeted NIR-PIT effectively interferes with the progression of EC and overcomes therapeutic resistance (Katsube et al., 2021). Combining the new CAF-targeted NIR-PIT with traditional anticancer drugs is expected to provide a more effective treatment strategy.

### Important Signaling Pathways

#### FGF/FGFR Pathway

Fibroblast growth factors are known to play a crucial role in regulating excessive development during the embryonic and adult stages of life. When FGF binds to FGFRS, the downstream Ras-MAPK, PI3K-Akt, and PLC-γ-PKC pathways are activated, inducing cell proliferation, differentiation, and tumor formation (Liu G. et al., 2021). Analysis of the ESCC gene database showed that FGF12 expression was elevated, meaning that it can be used as a biomarker (Bhushan et al., 2017). Analysis of ESCC samples also showed that the level of FGFR3IIIC, an FGF receptor, was elevated and tumor cell proliferation was increased (Ueno et al., 2016). In conclusion, systemic activation of the FGF/FGFR signaling pathway is important for the progression of EC. FGF/FGFR signaling plays a role in tumorigenesis, and a large number of drugs targeting this signaling pathway have been developed. Erdafitinib, a potent tyrosine kinase inhibitor of FGFR1/2/3/4, has been approved for the treatment of metastatic urothelial carcinoma (Loriot et al., 2019). Another FGFR inhibitor, pemigatinib, has also been shown to prolong survival in people with advanced cholangiocarcinoma (Abou-Alfa et al., 2020). A phase 2 clinical trial in EC patients using brivanib (FGF and VEGF inhibitors) showed an objective therapeutic effect on gastroesophageal cancer, but the data was insufficient to support the application of this drug in clinical treatment (Jones et al., 2019). Additional data is therefore needed to verify the effectiveness of FGF/FGFR inhibitors in EC.

#### HGF/c-Met Pathway

The binding of HGF to its high-affinity receptor, c-Met, can initiate the proliferation, migration, and angiogenesis of various tumors and promote tumor progression (Wang et al., 2020). HGF secreted by mesenchymal cells is also considered an important angiogenic factor. It binds to c-Met (mainly expressed in epithelial cells) exclusively and induces the activation of oncogenic pathways, angiogenesis, and scattering of cells, leading to metastasis (Ladeira et al., 2018). Additionally, upregulation of the HGF/c-Met signaling pathway can lead to activation of the β-catenin and PI3K/Akt pathways and deactivation of the E-cadherin pathway, promoting tumor invasion (Anderson et al., 2006). Several HGF/c-Met inhibitors which inhibit downstream signaling by either blocking HGF binding to c-Met or directly targeting c-Met are currently in clinical trials. Obinutuzumab and rilotumumab are humanized monoclonal antibodies that target HGF and inhibit its binding to c-Met (Shah et al., 2016; Catenacci et al., 2017). Unfortunately, using rilotumumab combined with cisplatin, capecitabine, and epirubicin as first-line treatment for MET-positive gastroesophageal adenocarcinoma did not give survival benefits (Catenacci et al., 2017). Obinutuzumab also failed in Phase III clinical trials. However, several c-Met inhibitors have been shown to be effective at inhibiting HGF/c-Met signaling by directly targeting c-Met. For example, AMG337, a highly selective small-molecule MET inhibitor, can effectively prevent c-Met/HGF binding. In a multi-center phase II study, AMG337, used as a single agent, showed significant anti-tumor activity in MET-amplified EAC patients (Van Cutsem et al., 2019). Additional data is needed to verify the effectiveness of HGF/c-Met inhibitors.

#### TGF-β Pathway

Many studies have clarified the important role of TGF-β in tumor regulation, including proliferation, angiogenesis, immune escape, and cell differentiation (Derynck et al., 2001). Interestingly, TGF-β plays a dual role in tumor progression, acting as a negative regulator in the early stage of tumor development but inducing epithelial mesenchymal transformation (EMT) and promoting migration in the late stages of development (Wojtowicz-Praga, 2003). Typically, TGF-β blocks the normal cell cycle in the G1 phase by inhibiting c-Myc and increasing the expression of P21 and P15, which are considered major regulators of the cell cycle (Wang L. et al., 2021). Activation of TGF-β/Smads inhibits the expression of cyclin-dependent kinase (CDK) inhibitors in advanced tumor cells and simultaneously activates the PI3K/Akt pathway, thereby preventing FoxO and Smad3 recombination. Ras/MAP kinases are also activated to induce EMT by bypassing TGF-β inhibition (Haque and Morris, 2017). As mentioned above, activation of the TGF-β signaling pathway can promote tumor progression and is therefore a potential therapeutic target. The most common inhibitors use the following mechanisms: (1) interferes with TGF-β synthesis, (2) blocks TGF-β signaling and downstream regulatory molecules, and (3) increases TGF-β endogenous or exogenous inhibitors. There are currently multiple TGF-β inhibitors in clinical trials. For example, Galunisertib, a small molecule inhibitor that directly targets TGFBR1 kinase, has shown satisfactory therapeutic effects in phase I clinical trials of advanced liver, pancreatic, breast, and colorectal cancers (Ahmadi et al., 2019). Unfortunately, TGF-β inhibitors have not been reported for EC. Moreover, TGF-β’s complex regulatory signals and dual effects also present challenges in its targeted therapy. However, TGF-β has shown anti-tumor effects in other cancer types, leading us to believe that it has great potential for use in the treatment of EC.

## Improving the Hypoxic Microenvironment

Hypoxia and acidosis are common phenomena in a variety of solid tumors, including EC, and lead to a series of physiological changes. Tumor cells rapidly proliferate and consume large volumes of oxygen. In addition, solid tumors compress blood vessels around the tumor and cause blood vessel blockages, which results in insufficient oxygen supply to the center of the tumor (Masoud and Li, 2015; Bhattarai et al., 2018). Normally differentiated cells rely mainly on the oxidative phosphorylation of mitochondria to provide energy for the cells, while most tumor cells depend on aerobic glycolysis, a phenomenon called Warburg effect (Vander Heiden et al., 2009). This phenomenon exacerbates hypoxia and lactic acid accumulation in solid tumors and promotes tumor metastasis. Under hypoxic conditions, the functional inactivation of prolyl-hydroxylase 2 (PHD-2) leads to reduced degradation of HIF-1, which is an important regulator of hypoxic microenvironments (Masoud and Li, 2015). Overexpression of HIF-1α upregulates GLUTs, hexokinase isoform 2 (HK2), pyruvate kinase isoform M (PKM), and other key factors, leading to tumor aerobic glycolytic metabolism (Kato et al., 2018; Sormendi and Wielockx, 2018; Han et al., 2020). The phenomenon is also applicable to EC, where several important regulatory factors such as HIF-1α, GLUT-1, and PKM2 have been found to be elevated (Xiaoyu et al., 2018). Another endogenous hypoxia marker, Carbonic anhydrase IX (CAIX), is also overexpressed in EC (Jomrich et al., 2014). In addition, the increased expression of HIF-1α can also directly upregulate VEGF and PD-L1, which are associated with tumor angiogenesis and immune escape, respectively (Augustin et al., 2020). In the hypoxic microenvironment, many immune cells are affected by HIF-1α, which reduces the immune response and promotes the proliferation of tumors. Moreover, hypoxia can also lead to genetic mutations that inhibit the effects of radiotherapy; downregulation of homologous recombinant proteins BRCA1 and BRCA2 in EC cells promoted G0-G1 cell cycle arrest and thus reduced the response to radiotherapy (Nguyen et al., 2013). A recent study using single-cell sequencing showed that HIF-1 expression decreased in paclitaxel-resistant EC cells, but this phenomenon was reversed with carfilzomib (Wu et al., 2018).

Hypoxia is one of the characteristics of solid tumors and is therefore a potential therapeutic target. Several approaches have been targeted at the hypoxic microenvironment, including HIF-1α targeted therapy, CAIX antagonists, nanomedicine, and traditional Chinese medicine (Wang et al., 2016; Wigerup et al., 2016; Chen et al., 2018; Li et al., 2018; Pan et al., 2018; Yu et al., 2018). Dihydroartemisinin, which is a derivative of artemisinin, can enhance the sensitivity of other treatments by inhibiting the expression of HIF-1α (Li et al., 2018). Chinese herbal medicine has thus proved unique in improving the hypoxic microenvironment and treating EC, but the specific underlying mechanism still requires in-depth analysis. EC can also be treated using phototherapy (Wang B. et al., 2021; Xiang et al., 2021). Recently, Liu J. et al. (2020) designed a cage-like carbon-manganese nanozyme which can not only improve the hypoxic microenvironment but also deliver a lot of photosensitizers to the tumor site, making it useful for real-time tumor imaging and enhancing the efficacy of phototherapy. This new nanomedicine has been verified in both *in vivo* and *in vitro* experiments. Improving the hypoxic microenvironment is an essential part of treating EC. At present, there are few treatment methods targeting the hypoxic microenvironment of EC and most of them are still in preclinical studies.

## Relationship Between Microenvironment Indicators and Prognosis

Multiple factors in the microenvironment play an indispensable role in EC development. Therefore, the detection of microenvironment indicators can predict the prognosis of patients to a certain extent. These prognostic indicators are summarized in Table 2.

**TABLE 2:** Factors and cells associated with poor esophageal cancer prognosis and their mechanisms of action.

Predictor	Mechanism	Expression	References
NF-κB	Induces the expression of IL-8 and IL-1β, intensifies inflammation, and inhibits tumor immunity	Up	Izzo et al., 2007, 2009
C-reactive protein	Directly reflect the degree of inflammation in the body	Up	Suzuki et al., 2020
Gram-negative bacteria	Its increase produces more lipopolysaccharides, which leads to increased inflammation and reflux	Down	Yang L. et al., 2012
IL-6	IL-6 activates downstream STAT3 expression after binding to its receptor. This allows tumor cells to survive in a highly toxic inflammatory environment	Up	Oka et al., 1996; Maeda et al., 2020
STAT3		Up	Hodge et al., 2005
IL-1β	It is two important pro-inflammatory cytokines, which promote tumor invasion and tumor-mediated immunosuppression	Up	Fitzgerald et al., 2002
IL-8		Up	Ogura et al., 2013
COX-2	COX-2 is an inflammatory enzyme responsible for the production of prostaglandin, which is associated with inflammation associated with gastrointestinal cancer	Up	Akutsu et al., 2011
MDSCs	MDSCs directly inhibits T cell activation, NK cell killing, and secretion of a large number of inflammatory cytokines to inhibit tumor immunity	Up	Chen et al., 2014
Tregs	Tregs may play a dual role in the occurrence of tumors, inhibiting inflammation in the early stage and inhibiting cytotoxic T cell function in the late stage, leading to immune escape	Up	Nabeki et al., 2015
M2 macrophage	Macrophages can differentiate into two completely different functional cell types: tumor-suppressing macrophages (M1) and tumor-promoting macrophages (M2). M1 macrophages play a role in tumor rejection, while M2 macrophages promote tumor progression	Up	Shigeoka et al., 2013
Th17	Th17 can directly or indirectly promote tumor growth. Th17 can express extracellular nucleotide enzymes CD39 and CD73, release adenosine, and inhibit CD8+T cells	Up	Chen et al., 2012
PD-L1	When they bind to PD-1, they inhibit T cell activation and promote immune escape	Up	Ohigashi et al., 2005
PD-L2		Up	
CAFs	CAFs secrete a variety of factors to promote tumor invasion and angiogenesis and promote immune evasion	Up	Underwood et al., 2015
TGF-β (Later stage)	TGF-β signaling appears to have a dual role in regulating tumorigenesis: in early stages it is a growth suppressor, but in later stages it promotes EMT and metastasis	Up	von Rahden et al., 2006
HGF	HGF induces the activation of oncogene signaling pathways by binding to its receptor c-Met and promotes tumor cell invasion and angiogenesis	Up	Takada et al., 1995
VEGFs	Trigger endothelial cell proliferation, migration, and breakdown of ECM to build new blood vessels	Up	Lord et al., 2003
MMP-2/7/9	Participate in extracellular matrix remodeling and promote tumor invasion	Up	Gu et al., 2005
CXCL12	The binding of its receptors CXCR4 and CXCR7 to tumor cells can induce tumor cell growth and promote angiogenesis and invasion	Up	Kaifi et al., 2005

## Conclusion

In this review, we summarized a variety of microenvironment-targeted therapies. At present, traditional therapies such as surgery, chemotherapy, and radiotherapy are still the main treatments for EC. Due to the drug resistance and side effects of traditional treatment, the current therapeutic effect does not meet our requirements. In a recent review, Luan et al. (2021) detailed the relationship between microenvironment and drug resistance in EC, suggesting that microenvironment-targeted therapy may be a breakthrough point for drug resistance. These results highlight the function of TME as a therapeutic target.

Some microenvironment-targeted drugs, such as PD-1/PD-L1 inhibitors and anti-angiogenesis drugs, have entered the clinic and shown good outcomes. New immunotherapies, such as CAR-T therapy, tumor vaccines, and oncolytic viruses, are undergoing clinical trials and have demonstrated initial therapeutic value. In addition, inhibition of the inflammatory microenvironment and improvement of hypoxia are also helpful for patient outcomes. However, existing treatment regimens have many limitations and are not sufficient to cure malignancies; thus, additional research is needed. First, enhancing the effectiveness of existing drugs, e.g., using biomarkers to identify drug-sensitive patients or combining drugs to enhance efficacy, is the simplest way of extending patient survival. Second, many microenvironment-targeted drugs that have shown significant anticancer effects in other tumors can also be used to treat EC. For example, TGF-β pathway inhibitors can not only directly inhibit the tumor but also enhance human immunity. Tests can be performed on EC to determine drug efficacy. Finally, in addition to the targets mentioned above, there are many mechanisms of the microenvironment that are currently unknown. Further studies of these mechanisms and active research and development of new drugs are important for achieving breakthroughs in EC. We believe that microenvironment-targeted therapy can achieve greater survival benefits for patients with EC and its specific mechanism requires further exploration.

## Author Contributions

YQ designed the study and reviewed the manuscript. LW and HH participated in study design and wrote the original draft of the manuscript. LW was mainly responsible for the design of the image. ZW, LS, and MY were involved in document retrieval and review. All authors agreed to the submission of the final manuscript.

## Conflict of Interest

The authors declare that the research was conducted in the absence of any commercial or financial relationships that could be construed as a potential conflict of interest.

## Publisher’s Note

All claims expressed in this article are solely those of the authors and do not necessarily represent those of their affiliated organizations, or those of the publisher, the editors and the reviewers. Any product that may be evaluated in this article, or claim that may be made by its manufacturer, is not guaranteed or endorsed by the publisher.
